# Simulation-based infection prevention and control training for medical and healthcare students: a systematic review

**DOI:** 10.3389/fmed.2025.1529557

**Published:** 2025-05-14

**Authors:** Akira Yoshikawa, Hiroyuki Ohtsuka, Keiichiro Aoki, Naonori Tashiro, Shusuke Togo, Kazuki Komaba, Satoshi Nogawa, Miwa Osawa, Megumi Enokida

**Affiliations:** ^1^Division of Morphology, Anatomy and Physiology, Department of Medical Basics, Specialty and Education, Showa Medical University, Graduate School of Nursing and Rehabilitation Sciences, Yokohama, Japan; ^2^Division of Neurological Science, Department of Rehabilitation, Showa Medical University, Graduate School of Nursing and Rehabilitation Sciences, Yokohama, Japan; ^3^Division of Occupational Therapy and Mental Health, Department of Rehabilitation, Showa Medical University, Graduate School of Nursing and Rehabilitation Sciences, Yokohama, Japan; ^4^Division of Cardiopulmonary Rehabilitation Science, Department of Rehabilitation, Showa Medical University, Graduate School of Nursing and Rehabilitation Sciences, Yokohama, Japan; ^5^Division of Healthcare Management, Department of Medical Basics, Specialty and Education, Showa Medical University, Graduate School of Nursing and Rehabilitation Sciences, Yokohama, Japan; ^6^Division of Clinical Engineering, Department of Medical Technology, Showa Medical University, Graduate School of Nursing and Rehabilitation Sciences, Yokohama, Japan; ^7^Division of Clinical Radiology, Department of Medical Technology, Showa Medical University, Graduate School of Nursing and Rehabilitation Sciences, Yokohama, Japan; ^8^Division of Health Science Education, Department of Medical Basics, Specialty and Education, Showa Medical University, Graduate School of Nursing and Rehabilitation Sciences, Yokohama, Japan

**Keywords:** infection prevention, infection control, infection education, simulation education, students, systematic review

## Abstract

**Introduction:**

Infection prevention and control education has traditionally been conducted in a lecture-based manner, and simulation-based educational strategies have become increasingly prevalent in the field of medical education in recent years. This systematic review aimed to compare the effectiveness of the simulation-based and traditional strategies of infection prevention and control education and to show the differences between these educational approaches. Furthermore, we identified the characteristics of simulation-based strategies for infection prevention and control education.

**Method:**

Systematic reviews and meta-analyses were performed according to the Preferred Reporting Items for Systematic Reviews and Meta-Analyses guidelines. A systematic literature search was conducted using the CENTRAL, MEDLINE, and Scopus databases for articles published between January 1990 and September 2022. This study focused on students enrolled in medical and health professional courses. As such, healthcare professionals already working in clinical settings, as well as kindergarten and elementary school students were excluded from the study. The quality of the included studies and the risk of bias in each study were assessed. A total of 254 articles were identified; 21 underwent secondary screening. Ultimately, 10 articles were selected for the final review.

**Results:**

Educational strategies between simulation- and lecture-based education showed improvements in knowledge acquisition. There was no significant difference in the rate of improvement between the two educational strategies. The characteristics of simulation-based educational strategies included confidence in skill performance, decision-making and problem-solving skills, emotional aspects related to infectious diseases (such as fear, empathy, self-reflection, and integration of complex information), and student satisfaction.

**Conclusion:**

This systematic review suggests that simulation-based education is effective in developing students’ skills and attitudes, while traditional lecture-based methods are more suited for reinforcing students’ knowledge. Therefore, it is essential to choose educational strategies based on specific learning objectives and outcomes.

**Systematic review registration:**

This systematic review protocol was preregistered in the Open Science Framework: https://osf.io/uj623/.

## 1 Introduction

The coronavirus disease 2019 (COVID-19) pandemic has caused fear of infection. It has resulted in a renewed awareness of the importance of hand hygiene and washing worldwide. Hand hygiene is one of the most common methods for preventing disease transmission (1). In addition to hand hygiene, healthcare professionals wear personal protective equipment (PPE) to treat patients and protect themselves from disease transmission and infection. Unfortunately, during the COVID-19 pandemic, a large number of healthcare professionals became infected and died (2, 3). Under these circumstances, it has become more important than ever to perform appropriate actions based on infection prevention and control and have appropriate education about infection, not only for healthcare professionals but also for students enrolled in medical, nursing, physical therapy, occupational therapy, and other related courses.

Participants in literature reviews of studies focusing on infection prevention and control education are often healthcare professionals (4–8). One reason for the need for infection prevention and control education for healthcare professionals is the prevention or reduction of healthcare-associated infections (HAIs; e.g. catheter-associated bloodstream infections, catheter-associated urinary tract infections, surgical site infections, and ventilator-associated pneumonia) and nosocomial infections (5, 6, 9). Although several studies on infection prevention and control education have focused on students, to the best of our knowledge, no systematic review reports exist on the results of these educational studies regarding infection prevention and control education. One report surveyed medical, dental, nursing, physical therapy, and occupational therapy students regarding their knowledge of infection prevention and control and their infection prevention measures during the COVID-19 pandemic. The report found that although these students had a high level of knowledge about infection prevention and control, they did not have sufficient ability to practice infection prevention and control actions, such as donning and doffing PPE and hand hygiene procedures (10). This means that students must acquire practical skills and knowledge during their student years before they work in clinical environments. Therefore, it is essential for the educational staff to develop evidence-based infection prevention and control strategies.

Simulation education is an educational strategy used to develop students’ practical skills. Simulation can be described as a continuum ranging from low-fidelity simulation (partial task trainers) to screen-based computer simulators, virtual reality (VR), role-playing (standardized patients), and high-fidelity simulation (full-scale simulation) (11). Simulation-based education strategies develop medical, nursing, and physical therapy students’ knowledge retention, clinical thinking, practical skills, confidence, and satisfaction compared to traditional strategies (12–15). Therefore, various simulation-based educational strategies can be adapted according to specific learning outcomes as well as educational and student levels. These systematic reports have described educational practices that mimic various clinical situations. However, the future challenges and limitations of simulation-based education primarily revolve around two key issues: (i) the significant costs involved in acquiring and maintaining advanced simulation equipment, and (ii) the need for educators to have the skills necessary to effectively utilize these simulators. Additionally, there is a requirement for the establishment of objective evaluation methods within simulation education (16). Specifically regarding objective evaluation in point (ii), organizations like the World Health Organization (WHO) have provided guidelines on proper procedures for putting on and removing PPE, as well as on hand hygiene. This presents an opportunity for objective evaluation in the field of infection prevention and control education through the use of checklists and other assessment tools within simulation-based education. However, our search for simulation-based education focused on practical training for infection control revealed that such training is predominantly offered to healthcare professionals in hospitals, with limited reports on its implementation in student education (17). Education on infection prevention and control requires the development of practical skills and knowledge. To effectively acquire both practical skills and knowledge, it is necessary to establish a simulation education strategy for infection prevention and control.

### 1.1 Aims

Our goal is to develop an infection prevention and control education strategy grounded in scientific evidence. To achieve this, the purpose of this study—serving as the first step—is to propose a simulation-based infection prevention and control education strategy. This approach is expected to effectively integrate students’ knowledge, skills, and attitudes, using systematic reviews and meta-analyses. The research questions for our systematic review and meta-analysis were as follows:

- Is infection prevention and control education based on a simulation strategy more effective than traditional educational strategies for students enrolled in medical, nursing, rehabilitation, and other related courses?
- What enhances learning satisfaction and the effectiveness of infection prevention and control education for students enrolled in medical, nursing, rehabilitation, and other related courses?

## 2 Methods

This systematic review was preregistered in the Open Science Framework Registry.^1^ This study followed the Preferred Reporting Items for Systematic Reviews and Meta-Analyses (PRISMA) guidelines (18). Additionally, a systematic meta-analysis was performed based on our previous protocol (17).

### 2.1 Study design

This study design was a meta-review of systematic reviews of articles on simulation and traditional infection prevention and control education for medical and healthcare students. Our study focused on articles published from 1990 to 2022. In a systematic review by Cant, R. P., it was reported that simulation education for medical and nursing students has been employed since 1999 (11). This suggests that simulation education has been used to teach students since the 1990s, and therefore, we have defined our study period as beginning in 1990. Systematic reviews included randomized clinical trials, pre-post designs, comparisons of two focus groups, or qualitative studies.

### 2.2 Participants

The participants were undergraduate and graduate students enrolled in medical and health-related occupational courses (doctors, dentists, nurses, physical therapists, occupational therapists, pharmacists, and other medical-related fields).

### 2.3 Intervention

Infection prevention and control education using simulation-based learning and/or training.

### 2.4 Comparison

Infection education using traditional education methods.

### 2.5 Outcome

Critical thinking, skill performance, knowledge acquisition, decision-making and problem-solving skills, self-efficacy, clinical reasoning skills, self-confidence, communication skills, teamwork, improved clinical performance, leadership skills, and student satisfaction.

### 2.6 Search strategy

We conducted a search of the CENTRAL, MEDLINE, and Scopus databases, with the last search date set to January 13, 2023. Our approach involved the use of a combination of text keywords and Medical Subject Headings (MeSH) terms tailored to each database. The concepts we aimed to capture included Patients: students who want to become healthcare professionals, “students,” “health occupations,” and “pupil nurses”; Intervention: simulation-based infection prevention and control education, “simulation training,” “infection”; Comparison: Comparison with traditional infection control education, “randomized,” and “clinical trials.” The details are shown in Table 1. Readers can search for the relevant articles by copying and pasting each search term listed in Table 1 in each database.

**Table 1:** Database search strategies and corresponding number of articles.

PubMed	Results	CENTRAL	Results	Scopus	Results
Students, Health Occupations[mh] OR student*[tiab] OR "pupil nurse*"[tiab] OR trainee*[tiab]	394,456	(student* OR "pupil nurse*" OR "trainee*"):ti,ab,kw	44,661	TITLE-ABS-KEY(student* OR "pupil nurse*" OR "trainee*")	1,506,053
Simulation Training[mh] OR (simulat*[tiab] AND (Education, Medical[mh] OR Education, Nursing[mh] OR Education, Dental[mh] OR Education, Pharmacy[mh] OR Education, Public Health Professional[mh] OR train*[tiab] OR learn*[tiab] OR teach*[tiab] OR educat*[tiab] OR Curriculum[mh] OR curriculum*[tiab] OR residenc*[tiab] OR patient*[tiab]))	131,738	[mh "Simulation Training"] OR (simulat* NEAR/4 (train* OR learn* OR teach* OR educat* OR curriculum* OR residenc* OR patient*)):ti,ab,kw	5,791	TITLE-ABS-KEY(simulat* W/3 (train* OR learn* OR teach* OR educat* OR curriculum* OR residenc* OR patient*))	83,805
Infections[mh] OR Infection Control[mh] OR infect*[tiab] OR ("ventilator associate*"[tiab] AND pneumonia*[tiab] OR coinfect*[tiab] OR communicab*[tiab] OR sepsis[tiab] OR transmit*[tiab] OR virus*[tiab] OR asepsis[tiab] OR steriliz*[tiab] OR disinfect*[tiab] OR bronchioliti*[tiab] OR antisepsis*[tiab] OR aerosol*[tiab] OR droplet*[tiab] OR airborne*[tiab] OR transmiss*[tiab] OR pathogen*[tiab] OR viral*[tiab])	5,345,341	[mh Infections] OR [mh "Infection Control"] OR (infect* OR ((ventilat* NEXT associate*) NEAR/4 pneumonia*) OR coinfect* OR communicab* OR sepsis OR transmit* OR virus* OR asepsis OR steriliz* OR disinfect* OR bronchioliti* OR antisepsis* OR aerosol* OR droplet* OR airborne* OR transmiss* OR pathogen* OR viral*):ti,ab,kw	227,039	TITLE-ABS-KEY(infect* OR ("ventilator associate*" W/3 pneumonia*) OR coinfect* OR communicab* OR sepsis OR transmit* OR virus* OR asepsis OR steriliz* OR disinfect* OR bronchioliti* OR antisepsis* OR aerosol* OR droplet* OR airborne* OR transmiss* OR pathogen* OR viral*)	8,214,216
#1 AND #2 AND #3	818	#1 AND #2 AND #3	73	#1 AND #2 AND #3	573
(controlled clinical trial[pt] OR randomized[tiab] OR randomised[tiab] OR clinical trials as topic[mesh:noexp] OR randomly[tiab] OR trial[ti] OR placebo[tiab]) NOT (animals[mh] NOT humans[mh])	1,434,505			(TITLE-ABS-KEY ({Clinical-trial} OR {controlled-trial} OR randomi* OR randomly OR (random W/4 (allocat* OR distribut* OR assign*)) OR {placebo} OR {trial} OR {groups} OR {subgroups} OR rct)) AND NOT (TITLE-ABS-KEY (animal AND NOT human))	6,489,633
#4 AND #5	71			#4 AND #5	109

### 2.7 Inclusion criteria

#### 2.7.1 Types of studies

We included both controlled clinical trials and randomized controlled trials (RCTs) in this review. Furthermore, we supplemented these with observational studies (including cohort and case–control studies) to obtain practical reports.

#### 2.7.2 Types of participants

The participants in the included studies comprised undergraduate and graduate students enrolled in medical and health-related occupational courses (medical doctors, dentists, nurses, physical therapists, occupational therapists, pharmacists, and other medical-related fields).

#### 2.7.3 Types of outcome measures

The following outcome measures were considered when including the studies: critical thinking, skill performance, knowledge acquisition, decision-making and problem-solving skills, self-efficacy, clinical reasoning skills, self-confidence, communication skills, teamwork, improved clinical performance, leadership skills, and student satisfaction.

### 2.8 Exclusion criteria

This was a systematic review of simulation-based infection prevention and control education for students. Therefore, studies involving healthcare professionals, non-medical and healthcare-related occupational course students, non-university students (e.g., kindergarten and elementary school students), and the general public were excluded. Additionally, we included articles published between January 1990 and September 2022, and excluded those published outside of this time frame.

### 2.9 Data collection and analysis

#### 2.9.1 Study selection

First, two investigators independently screened titles and abstracts using the text words and MeSH terms outlined previously in the initial literature search to determine whether articles potentially met the inclusion criteria. Articles that clearly did not meet the criteria were rejected. This double-blind study was conducted by two independent investigators using Rayyan software (19). In the primary screening phase, articles that did not match the review question were excluded based on an analysis of their titles and abstracts, and articles that could not be judged from their abstracts were retained. Next, the two reviewers independently reviewed the full texts of the remaining articles to determine their eligibility for review. In no case were the reviewers unable to extract all the required results for primary, secondary, and other outcomes from the included studies, and the authors were not contacted to explain the missing data in the studies. Disagreements at any stage were resolved through discussions between the two reviewers. If the reviewers failed to reach a consensus, a third reviewer was consulted for arbitration. The role of the third reviewer was to participate in discussions regarding any conflicts that arose. Before joining these discussions, the third reviewer independently reviewed the relevant articles. It is important to note that the third reviewer was not the final decision-maker. Instead, their role was to assist in reaching a consensus during the discussions.

#### 2.9.2 Data extraction and management

The data extraction sheet was piloted among the reviewers before extraction to ensure that it was easy for the eight reviewers to use. Subsequently, data were independently extracted by two reviewers, recorded, and managed using standard Microsoft Excel data recording spreadsheets by eight reviewers. Data were extracted to obtain a complete record of methodology, study design, participants, interventions, outcome measures, and results. Maximal data extraction was planned to ensure that the findings were adequately followed without returning to the original dataset. The data to be extracted conformed to Cochrane recommendations.

#### 2.9.3 Assessment of risk of bias

To assess the possible risk of bias (RoB) for each study, we evaluated and reported on the methodological RoB for the included studies on the following individual elements for RCTs: random sequence generation, allocation sequence concealment, blinding (participants, personnel), blinding (outcome assessment), completeness of outcome data, selective outcome reporting, similar baseline characteristics, and similar baseline outcome measurements. However, some non-randomized studies were reported. For these studies, we used the Risk of Bias in Non-Randomized Studies of Interventions (ROBINS-I), which aims to assess the RoB of estimates of the effectiveness or safety (benefit or harm) of interventions from studies that do not use randomization to allocate the intervention (20). In all cases, two reviewers independently assessed the RoB of the included studies, with any disagreements resolved through discussion or by consulting a third reviewer who was expected to be consulted previously for arbitration until a consensus was reached. We judged each item as high, low, with some concerns in RoB, and low, moderate, serious, critical, and no information in ROBINS-I, as mentioned in the criteria.

#### 2.9.4 Data synthesis and statistical analysis

Because statistical pooling was not possible, the findings from the quantitative and qualitative papers were consolidated into a single Microsoft Excel dataset with the following content:

- Author
- Population
- Country
- Type of study
- Student
- Intervention
- Comparison
- Outcome
- Key results

The diversity of the studies showed that a statistical meta-analysis was not feasible, so this review was presented using a narrative synthesis based on methodology. First, each investigator summarized the articles according to their theoretical perspective, using their individual knowledge and experience, and recorded this in an Excel sheet. This initial summary was done individually by each investigator. Next, the investigators collaborated to extract key themes relevant to the research questions from the appropriate papers and identified the content (21, 22).

## 3 Results

### 3.1 Characteristics of the study population (study selection)

**Figure 1:**
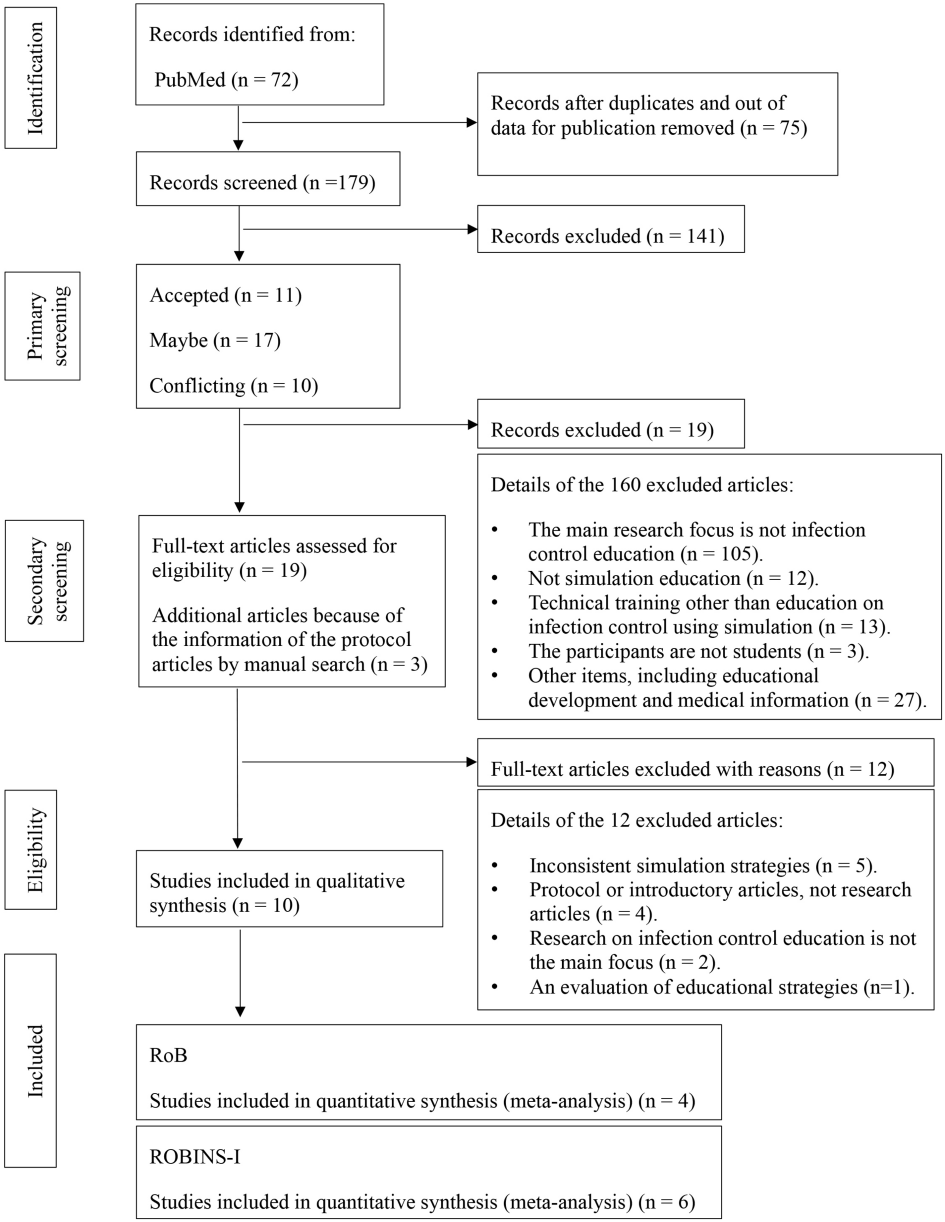
PRISMA flow chart.

In total, 72, 73, and 109 articles were extracted from PubMed, CENTRAL, and Scopus, respectively. Out of these, 75 were duplicates and outdated publications; therefore, 179 were eligible for primary screening. Primary screening was assessed by title, keywords, and abstract, resulting in 11 acceptable, 17 maybe, and 10 conflicting articles, which were discussed among two investigators, and 19 articles were selected. During this process, three articles were added by manual search; thus, 21 articles in total were screened by a secondary process. As a result of the secondary screening, 10 articles were eligible for inclusion (Figure 1).

### 3.2 Methodological quality assessment of intervention studies

**Figure 2:**
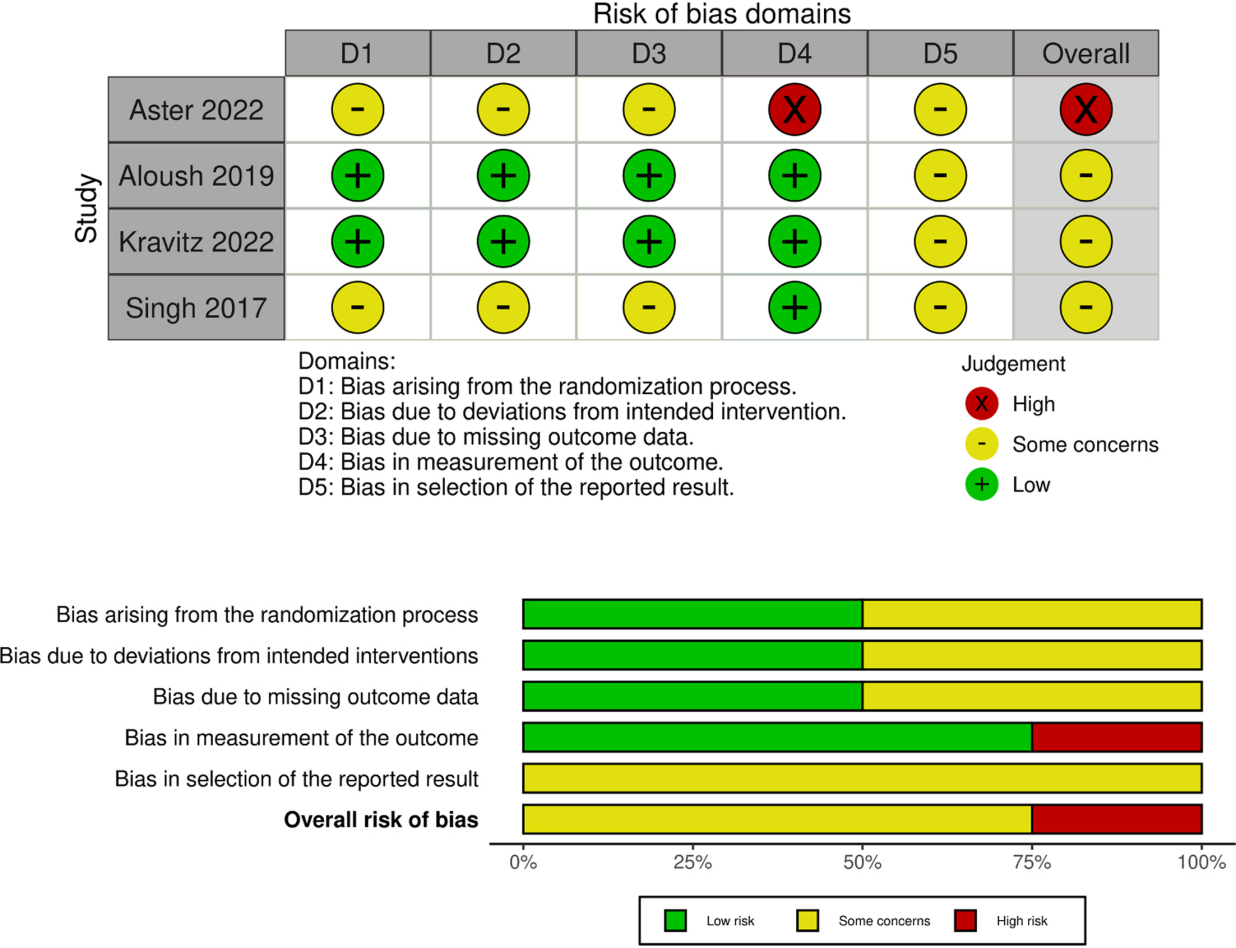
Risk of bias in included studies.

**Figure 3:**
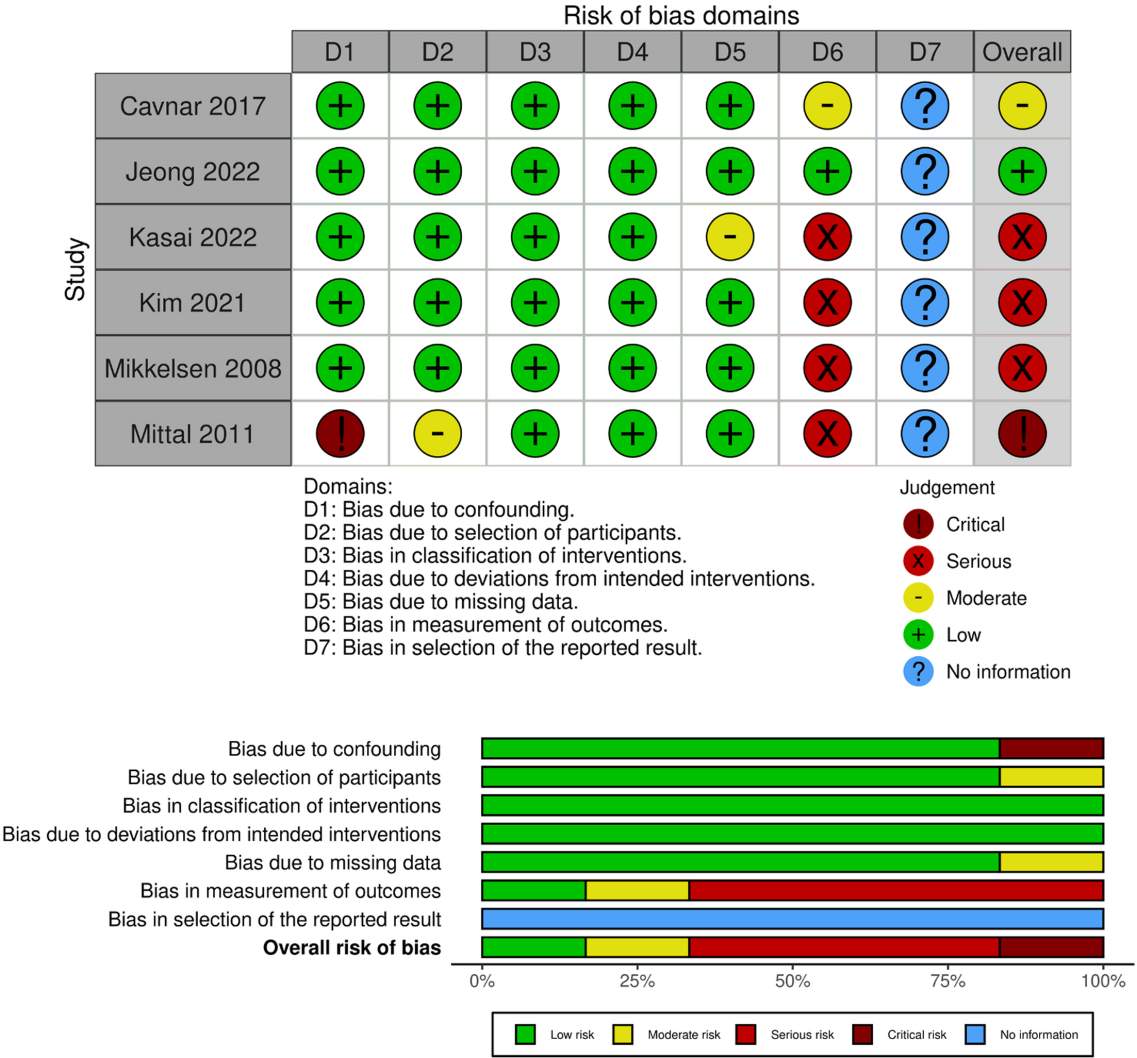
Risk of bias in non-randomized studies of interventions (ROBINS-I).

A study-level bias assessment was conducted for these articles. As four were RCT and six were non-RCT studies (pre-post or observational studies), the bias assessment was conducted using the RoB and ROBINS-I. In RoB, three out of four articles were rated as concerning, whereas the other resulted in a high risk (Figure 2 and Supplementary Figure 1). In ROBINS-I, one article was at low risk, one was at moderate risk, three were at serious risk, and one was at critical risk (Figure 3 and Supplementary Figure 2). These 10 articles showed the educational effectiveness of simulation-based infection prevention and control education for undergraduate and graduate students. A meta-analysis was not feasible in this study owing to substantial heterogeneity among the included studies in terms of study designs, interventions, and outcomes. Among the 10 studies included, four were RCTs, while the remaining six were non-RCTs, which included pre-post and observational studies. The methodological quality assessment indicated that three RCTs had “some concerns” regarding bias, while one had a “high risk” of bias. Among the six non-RCTs, three were classified as having a “serious risk,” and one was classified as having a “critical risk,” according to the ROBINS-I tool.

The simulation-based infection prevention and control education strategies varied significantly, encompassing not only those using low- and high-fidelity simulators but also VR, serious games, role-playing, and demonstrations as learning strategies. Additionally, the frequency, duration, and intensity of simulation training varied widely, contributing to the overall heterogeneity of the interventions.

Regarding outcome heterogeneity, a variety of assessment methods were employed across studies. For instance, one study utilized an objective structured clinical examination (OSCE) for technical assessment, while another applied a checklist based on Centers for Disease Control and Prevention guidelines or used its own developed assessment criteria. Interviews conducted revealed a diverse array of evaluation approaches. The combination of different study designs, the associated risk of bias, and varied outcomes further limited the feasibility of performing a meta-analysis.

### 3.3 Characteristics of research on infection prevention and control education using simulation for medical and healthcare students

Four studies conducted RCTs and six studies conducted non-RCTs, comparing simulation-based education before and after its implementation, or comparing it to traditional education. A total of 796 students participated in studies involving infection prevention and control education (Table 2).

**Table 2:** Description of the studies included in selected reviews.

Author	Number of participants	Country	Type of study	Students	Intervention	Comparison	Outcome	key results
Aster et al. (24)	100 fifth-year undergraduate medical students.	Germany	RCT	medical students	Students were randomly divided into three groups receiving different levels of exposure to virtual patients presenting with signs and symptoms of either infective endocarditis or community-acquired pneumonia in a serious game simulating an accident and emergency department.	No specific comparison group. Three groups were made for each level.	Game logfiles and an OSCE^*1^.	Higher exposure to virtual patients in the serious game did not result in superior OSCE^*1^ scores. However, there was good agreement between student performance in the OSCE^*1^ and game logfiles (r = 0.477, p = 0.005). An item response theory analysis suggested that items from the serious game covered a wider range of ability, thus better differentiating between students within a given cohort.
Aloush et al. (28)	131 nursing students	Jordan	RCT	Fourth-year nursing students.	Classroom lectures	Simulation course	Knowledge of central line-associated bloodstream infection prevention guidelines assessed using a structured 23-item questionnaire.	The overall knowledge scores in the pre-test were poor with no statistically significant difference between the two groups. In the post-test, both groups showed improvement in the majority of items. The participants in the classroom lectures group scored slightly higher in the majority of items in the post-test; however, there was no statistically significant difference in the overall scores.
Cavnar et al. (32)	Clinical laboratory sciences students assigned to either the intervention (*N* = 15) or control group (*N* = 12).	United State of America	Comparative study	Clinical laboratory sciences students	Students were educated by nursing faculty on careful hand hygiene when entering and exiting a patient’s environment. Moreover, students were instructed on the WHO six-step hand hygiene process, and rehearsed the steps.	Students were educated by nursing faculty on careful hand hygiene when entering and exiting a patient’s environment.	The intervention group demonstrated a significant and sustained increase in pre-patient hand hygiene times compared to 1the control group.	Pre- and post-simulation quizzes showed no significant difference in scores between the intervention and control groups (*p* = 0.588 for pre-scores; *p* = 0.756 for post-scores). Students’ actual hand hygiene was video recorded prior to entering the patient environment and again as they exited. The intervention group demonstrated a significant and sustained increase in pre-patient hand hygiene times compared to the control group.
Jeong et al. (31)	65 fourth-year students in nursing college.	Korea	Pre-post design	Nursing students	Developing a VR simulation program on COVID-19 and assessing the effectiveness of the program.	None	The experimental group showed a significantly higher learning satisfaction.	The experimental group showed a significantly higher learning satisfaction (t = 3.01, *p* = 0.004). Both groups presented statistically significant differences in knowledge on infectious respiratory diseases, self-efficacy and clinical reasoning between pre-test and post-test. However, knowledge (t = 0.47, *p* = 0.643), self-efficacy (t = 0.70, *p* = 0.944) and clinical reasoning were not different between the groups.
Kasai et al. (26).	82 fourth- and fifth-year medical students	Japan	Pre-post design	Medical students	Peer role-plays and a lecture on clinical education for COVID-19	None	Questionnaires and semi-structured focus group interviews.	Students’ satisfaction with COVID-19 education was high. No significant change was found among students concerning fear of COVID-19 before and after the program. The degree of burden of handling information on COVID-19 reduced significantly, whereas the degree with respect to the use of personal protective equipment (PPE), including appropriate wearing and removal of PPE and care of patients with confirmed COVID-19 while taking steps to prevent infection, exhibited a decreasing trend. The advantages of simulated clinical practice were segregated into five categories (infection prevention control, educational methods, burden on healthcare providers, self-reflection, and fear of COVID-19), and that of the lecture were segregated into four categories (information literacy, knowledge of COVID-19, educational methods, and self-reflection).
Kim et al. (29)	62 third-year undergraduate nursing students	Korea	Non-equivalent control group pre-post design	Nursing students	Lectures, skills training, and simulation using standardized practices.	Usual infection control education consisting of lectures, skills training, and peer tutoring practices.	Knowledge of standard precaution (the standard precautions knowledge measurement), awareness of standard precautions (The Awareness of Standard Precaution questionnaire), infection-related anxiety, and performance with infection control.	Both groups exhibited statistically significant increases in knowledge, awareness of standard precautions, and infection control performance after the intervention. Infection-related anxiety and infection control performance were significantly higher in the simulation using a standardized patient group. Both education programs influenced compliance with the standard precautions for infection control.
Kravitz et al. (23)	54 [medical students (*n* = 24) (44%) and emergency medicine and otolaryngology residents (*n* = 19) (35%)] participants	United States of America	RCT	Medical students and residents	VR-based PPE training	Five-minute instructional video and PowerPoint presentation	The primary outcome was the performance in donning and doffing PPE (assessed using a 64-point checklist). Secondary outcomes were participant preparedness and confidence level after training.	The VR group scored higher than the control group, but it was not statistically significant (VR: 55.4 vs. control: 53.3, *p* = 0.40) in the overall PPE score. Residents in the VR group performed significantly better than those in the control group (VR: 55.6 vs. control: 48.4, *p* = 0.009). VR participants reported higher levels of preparedness and confidence compared to control group participants. The VR group spent significantly more time in training than the control group (VR: 25.6 min vs. control: 6.5 min, *p* < 0.001).
Mikkelsen et al. (30)	141 nursing students	Kingdom of Norway	Comparative study in three groups	Nursing students	Simulation training (role-play: MRSA^*2^ and Norovirus) with four students and a teacher	Scenario-based (MRSA^*2^ and Norovirus) study groups consisting of 12 students and a teacher, study groups with 12 students and no teacher.	Scenario-based simulation training fits the way of “learning by doing.”	The findings indicated that scenario-based simulation training made the students more aware of how complex each scenario was. Events occurred that they had not expected, which led to a better recollection of details.
Mittal et al. (27)	64 s-year medical students and 21 residents	United States of America	Comparative study	Medical students	Germ simulation for teaching hand hygiene Principles and aseptic technique in urinary catheterization (UC)	Compared residents on UC skills	A task-specific checklist was used to assess UC skills.	Compared with residents, students washed their hands with equal effectiveness at baseline after simulation training, maintained better sterility, and had a higher technical proficiency score during UC. Students believed that it was a great idea to use simulated germs to highlight the effectiveness of hand washing and indicated that they would pay extra attention when washing hands.
Singh et al. (25)	102 medical students	India	RCT	Second-year medical undergraduate students.	Low-fidelity simulation in the form of VSG and CDG	The control group was not exposed to any of the low-fidelity simulations in the form of case discussions and video demonstrations	The primary outcome was the TEQ^*3^, and the secondary outcome was the infection control module and knowledge test.	No significant difference in pre-test TEQ^*3^ scores (*p* = 0.87). Post-test TEQ^*3^ scores showed a significant difference between the groups (*p* = 0.026), with both VSG^*4^ (*p* = 0.046) and CDG^*5^ (*p* = 0.011) scoring higher than the control group. Significant difference in performance among the groups (*p* = 0.016) in knowledge test scores. Post-hoc analysis showed that VSG^*4^ performed significantly better than the control group (*p* = 0.005), whereas CDG^*5^ showed similar performance for the control group (*p* = 0.074). General perceptions: over 90% of students felt the module was well-planned, relevant, and useful. Both VSG^*4^ and CDG^*5^ helped students understand the impact of HAIs on patients and their families. CDG^*5^ was found to be more stimulating than VSG^*4^.

*1 OSCE: Objective Standardized Clinical Examination; *2 MRSA: Methicillin-Resistant *Staphylococcus aureus*; *3 TEQ: Toronto Empathy Questionnaire; *4 VSG: Video Show Group; *5 CDG: Case Discussion Group.

Of the 10 articles in this review, five were aimed at medical students (23–27), four at nursing students (28–31), and one at clinical laboratory students (32). The definition of a simulation-based educational strategy varied across the literature: two studies used VR (23, 31), five studies used role-play or demonstrations (26, 27, 29, 30, 32), one study used low-fidelity simulators (25), one study used high-fidelity simulators (28), and one study used serious games (24).

Regarding infection prevention and control educational topics, four articles were related to the donning and doffing of PPE and hand hygiene (23, 29, 31, 32), three articles were related to HAIs (25, 27, 28), and three articles were related to the pathophysiology of infectious diseases (24, 26, 30).

To compare the educational effectiveness of simulation-based infection prevention/control education with traditional classroom lectures and viewing of video materials, knowledge tests, practical tests (such as the objective, standardized clinical examinations), and questionnaires were used as evaluation methods. However, the detailed content of these evaluation items depended on the individual, and there were no common evaluation items.

Although students’ knowledge test scores improved after both simulation and traditional education, most studies found no significant differences between these two educational strategies. The benefits of simulation education were confidence in skill performance (23, 27, 32), decision-making and problem-solving skills (25, 27, 28), emotional aspects related to infectious diseases (fear, empathy, self-reflection, integration of complex information) (25, 26, 29, 31), and student satisfaction with simulation-based infection prevention and control education (26, 31). A summary of this is presented in Table 3.

**Table 3:** Thematic summary of the characteristics of simulation-based strategies.

Type of Simulation	Author	Educational challenges	Difference in knowledge acquisition compared simulation to traditional education method	Advantage of simulation education
Role-play or demonstrations	Cavnar et al. (32)	Education on hand hygiene techniques for clinical laboratory sciences students.	Not significant	The duration of hand hygiene has increased.
	Kasai et al. (26)	Education on clinical practice regarding COVID-19 for medical students.	Not significant	Students’ satisfaction was high. The advantages of simulation-based education include infection prevention measures, educational methods, the burden on healthcare providers, self-reflection, and fear of COVID-19.
	Kim et al. (29)	Education on infection control for nursing students.	Not significant	Infection-related anxiety and infection control performance were significantly higher.
	Mikkelsen et al. (30)	Education on cross-infections based on MRSA and norovirus for nursing students.	Not compared	Scenario-based simulation training fits the way of “learning by doing.” Providing “awareness” to students.
	Mittal et al. (27)	Education on hand hygiene techniques for medical students.	Not compared	The students’ proficiency in skills is enhanced.
VR	Jeong et al. (31)	Development of VR scenarios for COVID-19 education targeted at nursing students.	Not significant	Students’ satisfaction was higher. Students’ self-efficacy and clinical reasoning skills are enhanced.
	Kravitz et al. (23)	Education on the proper donning and doffing of PPE targeted at medical students.	Not compared	Increased confidence in donning and doffing PPE.
Low-fidelity simulation	Singh et al. (25)	Education on healthcare-associated infections (HAIs) targeted at medical students.	Simulation > traditional	Increased empathy and knowledge regarding HAIs.
High-fidelity simulation	Aloush et al. (28)	Education on central line-associated bloodstream infection (CLABSI) targeted at nursing students.	Not significant	Increased clinical skills and decision-making.
Serious games	Aster et al. (24)	Infection control education for medical students focusing on endocarditis and community-acquired pneumonia.	Not compared	Complementing student performance.

## 4 Discussion

This systematic review compared the educational effectiveness of the simulation-based traditional education strategies for infection prevention and control and showed the differences between these educational approaches. Furthermore, we identified the characteristics of simulation-based strategies for infection prevention and control education.

During the COVID-19 pandemic, nursing students reported feeling a strong desire to help others; however, they were conflicted by their lack of knowledge and skills related to infection prevention and control, as well as their own fear of infection (33, 34). Furthermore, reports on the knowledge and skills of nursing students regarding hand hygiene indicated that, although they have higher infection-related knowledge than general students, they have significantly less clinical experience than practicing nurses in clinical situations. This lack of experience increases the risk of infection in clinical practice (35). These reports highlight the deficiency in infection prevention and control education prior to clinical practice. Regarding students’ knowledge and behaviors related to infection control and prevention, several articles reported that medical students have low awareness of hand hygiene (36). Evaluations of hand hygiene knowledge among students in medical, dental, and nursing schools revealed that their knowledge level was low-to-moderate (37), and female students had a higher awareness of hand hygiene practices than male students (38). These reports reveal the current state of infection prevention and control knowledge and practical skills of the students. Hence, the importance of infection prevention and control education for students has been reaffirmed, and there is a recognized need to provide scientific information to educational staff on infection prevention and control educational strategies and their effectiveness. These aspects had been previously noted in the field of medical education, prior to the COVID-19 pandemic. However, the COVID-19 pandemic has led to the identification of more specific issues within medical education settings, such as those pertaining to infection prevention and control education, related to PPE (23, 26, 31). Personal protective equipment techniques are considered one of the most basic infection control education skills. However, this systematic review suggests that the COVID-19 pandemic has significantly increased the importance of ensuring that students acquire such PPE knowledge and skills. Additionally, simulation-based infection prevention and control education focused on awareness of the COVID-19 pandemic led to a decrease in anxiety in some students, while anxiety increased in others (26). Confronting the global fear of COVID-19 without the correct knowledge increases fear among students. A suggested method to alleviate this fear is the acquisition of accurate pathological knowledge alongside the necessary PPE accurate techniques (29, 39). These findings suggest that, through this systematic review, infection prevention and control education using simulation as an educational strategy may have been transformed by the COVID-19 pandemic into more specific learning content, such as pathological knowledge of COVID-19, and donning and doffing PPE, including the hand hygiene techniques, as a way to protect oneself when working with patients.

The WHO guidelines for infection prevention and control educational strategies for medical students suggest a combination of the following methods: (1) lectures and clinical placements, (2) online activities, (3) on-the-ward activities, (4) small group tutorial teaching, (5) problem-based learning, (6) simulation/skills laboratories, and (7) traditional tutorials.^2^ Among these educational strategies, we conducted a systematic review to compare traditional educational methods with simulation-based education, which has recently been recognized for its effectiveness. For undergraduate and graduate students enrolled in medical and healthcare-related occupational courses, simulation-based education has several benefits: (1) improved knowledge and skills, (2) a safe learning environment, (3) enhanced clinical judgment and decision-making, (4) increased self-confidence and satisfaction, (5) effective non-technical skills training, (6) reduced anxiety and increased confidence in technical skills, and (7) long-term retention (12, 40–42). However, simulation-based educational strategies face challenges such as high costs and resource intensiveness (requiring substantial resources, including dedicated space, equipment, and trained faculties) (42, 43).

The results of this systematic review indicate that the characteristics of simulation-based educational strategies included confidence in skill performance, decision-making and problem-solving skills, emotional aspects related to infectious diseases (such as fear, empathy, self-reflection, and integration of complex information), and student satisfaction. However, studies that utilized simulation education strategies also had their limitations. In strategies using VR, technical challenges in creating scenarios were noted (31). Furthermore, students participated only in brief or single-session VR scenarios, which resulted in limited interpretations of the evaluation outcomes concerning knowledge, skills, and attitudes (23, 31). In role-play strategies, several concerns have been raised, including the small number of participants and potential bias in participant recruitment (27, 29, 32). Additionally, there may be cultural bias present in the region where the study was conducted (26), and doubts regarding the generalizability of infection control education arising from research limited to a specific medical department (26). The simplicity of the infection control scenario also raises questions about whether the results can be applied to other infection control situations (29). The requirement for substantial human resources to conduct simulation education has also been emphasized (30). In educational research using low- and high-fidelity simulators, similar concerns have been noted, such as small sample sizes and potential cultural bias related to the study’s location. There is also a limitation in the focus solely on knowledge and skills, without a detailed analysis of the factors that enhance them (28). Additionally, the need for evaluation metrics that closely align with clinical practice has been highlighted. There is uncertainty about whether assessments conducted in simulation settings effectively translate to actual practice (25). In educational strategies using serious games, several issues have been identified, including participant drop-out, limited interpretation of results owing to the implementation being similar to VR in single sessions, and the need for more appropriate evaluation methods (24). It is essential to verify whether students are indeed capable of practicing their skills and demonstrating the appropriate attitudes, as questionnaire evaluations often rely on self-assessment (26).

To facilitate a comparison between traditional infection control education and the new educational strategy of simulation-based infection control education, we focused on articles published from 1990 to 2022. In our investigation, the earliest report on simulation-based infection control education was found to be from 2008. Regarding simulation education across all medical education, not just focused on infection prevention and control, McGaghie et al. have reported that various aspects of simulation education—including (i) fidelity, (ii) proficiency-based learning and outcome measurement, (iii) instructor training, (iv) curriculum development, and (v) the overall growth and maturation of instructor training—have significantly advanced over time (44). The strategy of simulation-based education helps students develop their skills and knowledge while gaining experience in a relatively safe and controlled environment. Simulation is recognized for providing a safe and relevant learning experience by operating “ex vivo,” meaning it does not involve real patients. However, a challenge remains in determining whether students who have undergone simulation education and acquired skills, knowledge, and attitudes can effectively apply these competencies in clinical practice (45). Simulation education is considered an effective method owing to the secure learning environment it creates. However, it can sometimes lead to a disconnect from real clinical settings, which may create a sense of deception for students (46). Therefore, educators must present clear learning objectives when conducting simulation education and provide thorough explanations for each scenario (44). It is also essential for educational research to investigate whether students’ knowledge, skills, and attitudes gained through simulation education are effectively utilized in clinical situations. Additionally, to implement simulation education successfully, educators may need to participate in seminars, such as Technician Training Programs, to learn about effective simulation strategies (47). Participation in such programs involves financial considerations, and the costs associated with acquiring and maintaining high-fidelity simulators can far exceed these expenses. To maximize educational effectiveness within limited resources, it is crucial to identify the specific benefits of simulation education and develop programs that enable students who have experienced simulation to fully realize their capabilities in clinical practice (48).

This systematic review indicated that traditional educational strategies, such as lectures, remain important in the acquisition of knowledge related to infection prevention and control. The integration of knowledge, skills, and attitudes is essential in both medical and general education (49). This suggests that, in infection prevention and control education, it is crucial to determine educational strategies based on specific educational objectives and learning outcomes. In light of these considerations, the necessity for standardization of education regarding the essential knowledge, as well as skills of donning and doffing PPE and hand hygiene has been supported (50). We propose two key initiatives to establish standardization in this area of education. Our primary challenge is the development of standardized evaluation methods. While various assessment techniques were used in this study to assess educational effectiveness, the significant variability in these evaluation methods prevented us from synthesizing and validating the findings across the individual studies. As a specific approach to simulation education in infection control, we recommend utilizing checklists based on WHO guidelines for hand hygiene^3^ and PPE.^4^ Additionally, we suggest developing knowledge assessment tests for students that are derived from these guidelines for hand hygiene and/or PPE checklists. Standardizing educational intervention protocols and adopting consistent outcome measurement criteria will allow for the implementation of high-quality RCTs. This method will facilitate future meta-analyses, improve researcher comparability, and contribute to identifying superior educational methods. Moreover, we propose conducting mid- and long-term outcome evaluations to determine whether students who have experienced simulation education can apply what they learned in clinical situations afterward. While many studies on simulation education focus on short-term outcomes, we, as educators, expect students to integrate their knowledge, skills, and attitudes into their clinical practice. Therefore, it is essential to assess changes in students’ behavior not only in the short term but also in the mid- and long term. Based on the results of this systematic review, we believe that effectively combining simulation with traditional lectures as educational strategies for infection prevention and control education will lead to the development of enhanced educational approaches.

### 4.1 Limitations

The evaluation of educational outcomes varied across studies; even when quantified, the assessed evaluation scores or items differed, and qualitative aspects such as interviews with students were also included. Consequently, integration between studies is not feasible, and future research will need to establish standardized metrics to indicate the effectiveness of infection prevention and control education. Additionally, it is important to consider the potential publication bias in educational research, as many studies have yielded positive results (51).

## 5 Conclusion

This systematic review aimed to establish a foundation for infection prevention and control educational strategies by comparing the effectiveness of simulation-based strategies and traditional educational strategies, specifically for undergraduate and graduate students enrolled in medical and healthcare-related occupational courses. Although both simulation and traditional education improve student knowledge, most studies found no significant differences between these two teaching strategies. The benefits of simulation education include confidence in skill performance, decision-making and problem-solving skills, emotional aspects related to infectious diseases, and student satisfaction with simulation-based infection prevention and control education. Infection prevention and control education require students to integrate knowledge, skills, and attitudes to develop practical competencies. This includes the use of simulation-based education, which excels in the acquisition of skills and attitudes alongside traditional lectures, which are effective for reinforcing knowledge.

## Data Availability

Data that support the findings of this study are available from the corresponding author upon request.

## Footnotes

^1^ https://osf.io/q27cj/

^2^ https://www.who.int/publications/i/item/9789241598316

^3^ https://www.who.int/publications/i/item/9789241597906

^4^ https://www.who.int/teams/health-product-policy-and-standards/assistive-and-medical-technology/medical-devices/ppe
