# Supplementary material for: Simulation-based infection prevention and control training for medical and healthcare students: a systematic review
Source: Front Med (Lausanne). 2025 May 14;12:1529557. doi: 10.3389/fmed.2025.1529557 (PMC12116496; doi:10.3389/fmed.2025.1529557)
Supplement: Supplementary file 1 [file Data_Sheet_1.PDF]

Supplementary Material

1 Supplementary Figures

Supplementary Figure 1. Explanation of “Some Concerns” and “High Risk” in the Assessment Results of Risk of Bias (RoB)

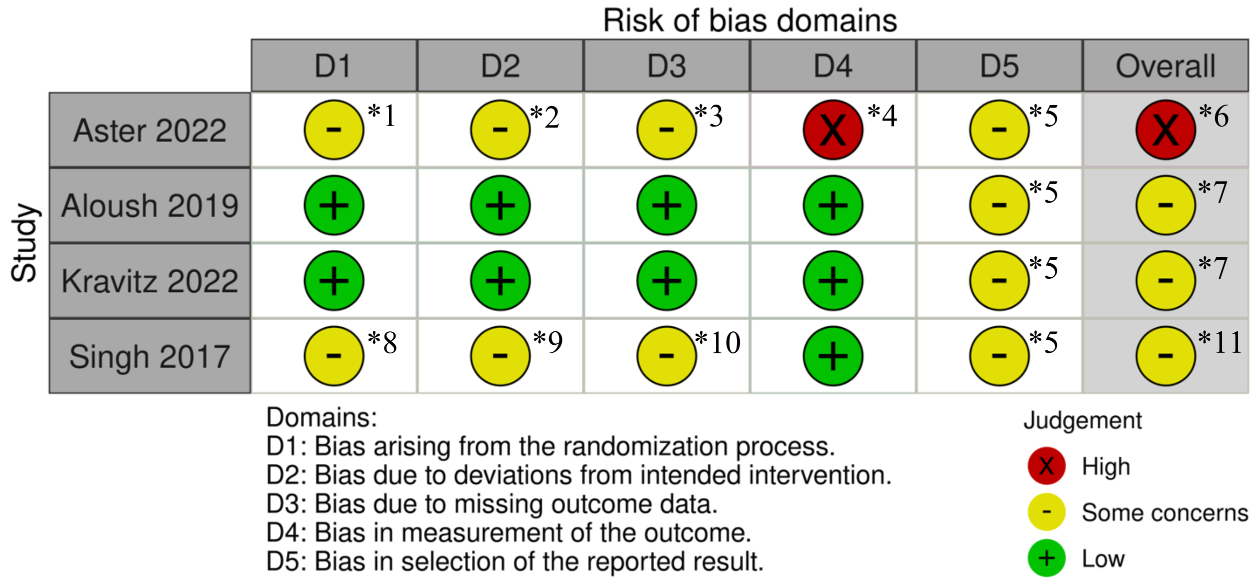

*1: The method of allocation sequence is not clearly specified.

*2: Although an intention-to-treat (ITT) analysis was not conducted, it is not believed to have a significant impact on the study results.

*3: There are deviations exceeding 5% during the study, and an analysis to correct for these deviations has not been conducted. However, it is unlikely that these deviations depend on the true values.

*4: There is no information regarding whether the outcome assessors were aware of the interventions received by the participants, and there is no clear evidence that this lack of information has not influenced the evaluation results.

*5: There is a lack of pre-study information, such as a protocol paper.

*6: Because the assessment in Domain 4 was rated as “High risk.”

*7: Because the assessment in Domain 5 was rated as “Some concerns.”

*8: Although random allocation was performed using block randomization, there is no information regarding the allocation order, and no baseline imbalances were observed.

*9: Because the participants were aware of which group they were assigned to.

*10: Although missing data is present, the reasons for it are not clearly stated.

*11: Because the assessments in Domains 1, 3, and 5 were rated as "Some concerns."

Supplementary Figure 2. Explanation of "Moderate," "Serious," and "Critical" in the Assessment Results of Non-Randomized Studies of Interventions (ROBINS-I)

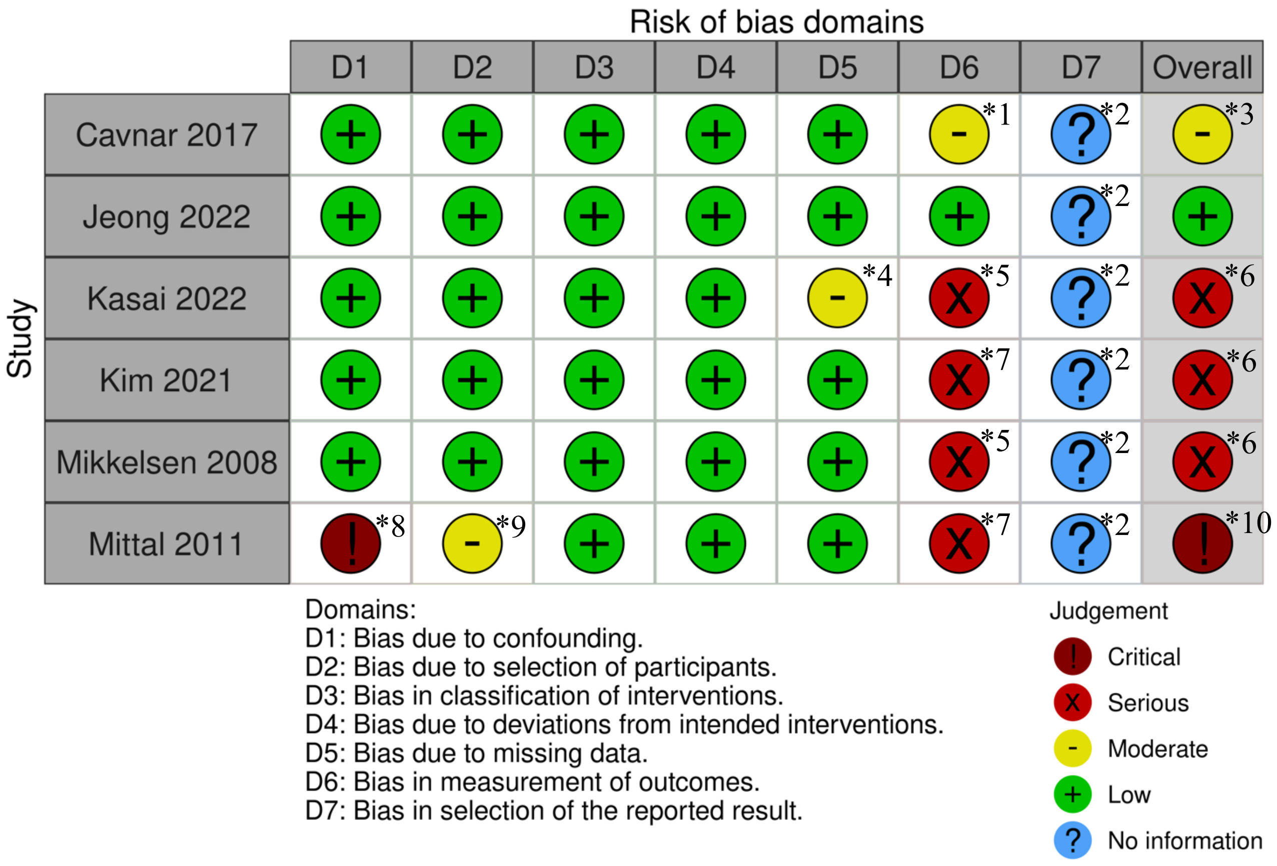

*1: Although the students were aware of which group they were assigned to, it was considered that this would have a negligible impact on the outcome results.

*2: Due to the lack of information, such as a protocol paper, there are no details available regarding the selection of outcomes.

*3: Because the assessment in Domain 6 was rated as "Moderate."

*4: Initially, 82 participants were targeted, but 7 were excluded (9%). Additionally, during the interviews, there were 74 participants, with 1 case of missing data for an unknown reason.

*5: One of the outcomes involved a semi-structured interview, which included subjective elements.

*6: Because the assessment in Domain 6 was rated as "Serious."

*7: The outcome assessors were aware of the participants' interventions while conducting their evaluations.

*8: Participants were selected based on clear confounding factors, such as educational background and clinical experience.

*9: Participants were not selected after the intervention began; however, it is known that a clear imbalance emerged between the experimental and control groups from that point onward.

*10: Because the assessment in Domain 6 was rated as “Critical.”
